# Microwave-Assisted Hydrothermal Processing of *Rugulopteryx okamurae*

**DOI:** 10.3390/md21060319

**Published:** 2023-05-25

**Authors:** Tania Ferreira-Anta, Noelia Flórez-Fernández, Maria Dolores Torres, José Mazón, Herminia Dominguez

**Affiliations:** CINBIO, Universidade de Vigo, Department of Chemical Engineering, Facultade de Ciencias, Campus Ourense, As Lagoas, 32004 Ourense, Spain

**Keywords:** *Rugulopteryx okamurae*, microwave, sea water, bioactive compounds, biobased materials

## Abstract

One possible scheme of *Rugulopteryx okamurae* biomass valorization based on a green, rapid and efficient fractionation technique was proposed. Microwave-assisted pressurized hot water extraction was the technology selected as the initial stage for the solubilization of different seaweed components. Operation at 180 °C for 10 min with a 30 liquid-to-solid ratio solubilized more than 40% of the initial material. Both the alginate recovery yield (3.2%) and the phenolic content of the water-soluble extracts (2.3%) were slightly higher when distilled water was used as solvent. However, the carbohydrate content in the extract (60%) was similar for both solvents, but the sulfate content was higher for samples processed with salt water collected from the same coast as the seaweeds. The antiradical capacity of the extracts was related to the phenolic content in the extracts, but the cytotoxicity towards HeLa229 cancer cells was highest (EC_50_ = 48 µg/mL) for the extract obtained with distilled water at the lowest temperature evaluated. Operation time showed a relevant enhancement of the extraction performance and bioactive properties of the soluble extracts. The further fractionation and study of this extract would be recommended to extend its potential applications. However, due to the low extraction yield, emphasis was given to the solid residue, which showed a heating value in the range 16,102–18,413 kJ/kg and could be useful for the preparation of biomaterials according to its rheological properties.

## 1. Introduction

*Rugulopteryx okamurae* (*Dictyota marginata*, *Dilophus marginatus*, *Dictyota okamurae*, *Dilophus okamurae*, Dictyotales, Phaeophyceae) is a brown seaweed native from the warm temperate western Pacific Ocean [1], which in recent years colonized the southwestern coasts of Europe [2] and became an aggressive invader [3,4,5]. The Strait of Gibraltar is an area exposed to multiple environmental changes including other invasive seaweed species, but in this area *R. okamurae* has displaced the local biota and substantial amounts of beach coast material, generating a great ecological impact and also affecting human and economic activities [3]. The urgent need to implement management measures has been highlighted [6], and solutions integrated into a circular economy model are preferred [7].

This high invasive potential has been ascribed to the chemical defenses, especially to diterpenoids [8]. Dilkamural, a compound with deterrent properties and harmful effects on generalist herbivores, can be found at high concentrations in this seaweed and was not described previously in the invaded area [9]. The diterpenoid dilkamural, obtained by chromatographic purification of an ethyl acetate extract of this alga, showed antimicrobial activity [10]. Other terpenoids, rugukadiol A, rugukamurals A–C, and ruguloptones A–F, have been identified [11]. Among secondary metabolites, the terpene and polyphenolic compounds stand out, presenting photoprotective, antioxidant, and immune-stimulant properties [12]. Different solvent extracts from *Rugulopteryx okamurae* have shown selective cytotoxicity to human leukemic cells [13], with inhibitory effects on α-glucosidase, but were non-cytotoxic on mouse pre-adipocytes cells [14]. However, data on the biomedical potential are scarce, and only antibacterial activity and anti-inflammatory have been reported [11]. 

The extremely rapid expansion of this brown seaweed in coasts of Southwest Europe has been compared to that of *Sargassum* sp. [15] and the future impact of such tides could be quite different if they become regarded as potential crops rather than harmful weeds [16]. The biomass valorization has been proposed to lower the expansion of this invasive species; De la Lama-Calvente et al. (2021) proposed an anaerobic co-digestion of *R. okamurae* biomass with olive mill solid waste to enhance both the methane yield and biodegradability of both substrates [15] and De la Lama Calvente et al. (2023) proposed a new mechanical pretreatment with zeolite and a thermal pretreatment at 120 °C for 45 min to improve the process [7]. A microwave irradiation pretreatment before enzyme hydrolysis for obtaining reducing sugars was successful to produce volatile fatty acids in dark fermentation [17]. Santana et al. (2022) developed bio-based plastic materials, processed by injection molding blends of the raw seaweed with glycerol to obtain environmentally friendly materials [18]. Patón et al. (2023) proposed traditional composting and black soldier fly larvae composting to eliminate algae, providing fertilizers and animal proteins, because toxins in the algae do not affect the long-term survival, growth, or reproduction of these invertebrates [19]. Alternatives based on the cascade fractionation of this resource have not been addressed, although this biorefinery approach to obtain fertilizers, bioactive compounds, and biofuels proved technically and economically feasible for *Sargassum* biomass [20,21]. Later authors indicated a wide range of potential applications of *Rugulopteryx okamurae* key components such as fucoidans and phenolic compounds for the development of pharmaceutical, cosmetic, and nutraceutical products. The corresponding alginates could be employed from food packaging to wound dressing.

The main aim of this work is to propose one of the multiple possibilities for an integral cascade valorization sequence of the invasive brown seaweed *Rugulopteryx okamurae* biomass following a biorefinery approach. The major novelty is the preliminary exploration of the extraction of the alginate, sulfated oligosaccharides and phenolic compounds as well as the formulation of printable alginate-based biomaterials using the residual solids after hydrothermal treatment.

## 2. Results and Discussion

### 2.1. Raw Material

The dried algae contained 8.06 ± 0.16% moisture and the proximal composition in dry basis is shown in Table 1. The major fractions were protein (16.43%) and carbohydrates, the most abundant constituent being glucose (11.69%), followed by galactose (2.76%), fucose (6.38%), mannose (1.91%), uronic acids (1.65%), and xylose (0.68%).

Mercado et al. (2022) reported a highly variable N content in *R. okamurae* ranging from 1.4% to 4.5%, suggesting that this species has high N storage capacity that is potentially usable when the external N concentration decreases [22]. This storage capacity of N is a common feature of bloom-forming algae, which allows them an opportunistic growth when the external conditions are favorable.

A relatively high lipid content (6.2%) was observed, in relation to the values found for other brown seaweeds. The fatty acid profile was composed of palmitic acid (50.1%), myristic acid (22%), 9-hexadecenoic acid (11%), 9-octadecenoic (12%), stearic acid (2.6%), and eicosanoic acid (2.3%).

The solvent used for the extractives’ quantification had a notable impact on the results: those recovered after ethanol and the mixture of methanol, acetone, and water exhibited higher values (above 11%) than those treated with hexane (1.7%).

Brown seaweeds contain polysaccharides (alginates, laminarin, and fucoidan), phlorotannins, terpenoids, minerals, vitamins, and fatty acids. No information on the sulfated polysaccharides of this seaweed has been found in the literature.

### 2.2. Microwave-Assisted Extraction

**Figure 1:**
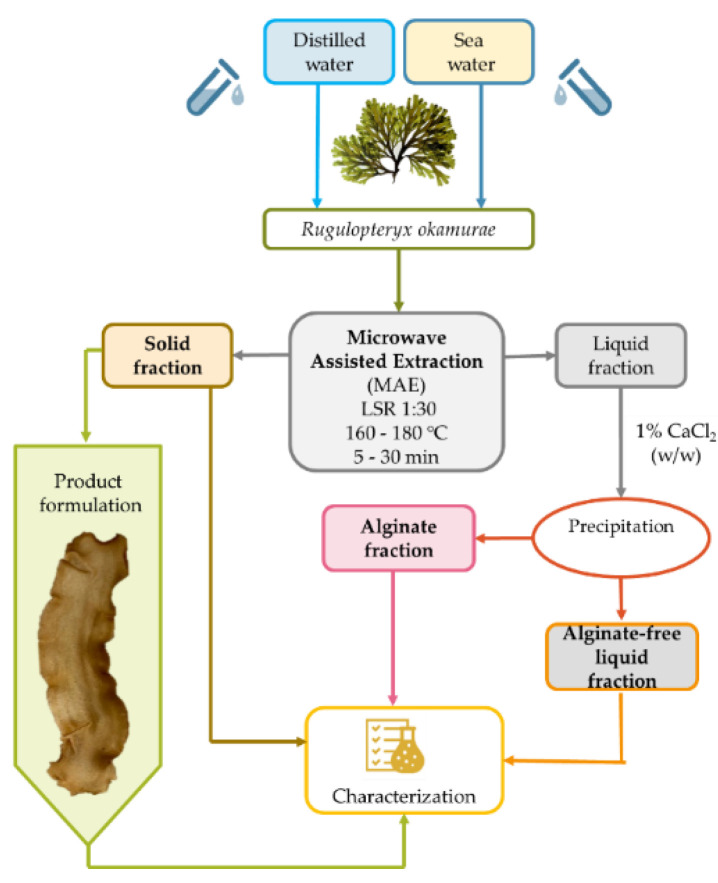
Flow diagram of the proposed sequence for the microwave-assisted hydrothermal fractionation of *Rugulopteryx okamurae* biomass.

The aqueous extraction operating under pressurized conditions with microwave heating can be proposed as a first stage for the fractionation of *R. okamurae* biomass (Figure 1). This approach has previously been selected for the extraction of bioactive compounds from other brown seaweeds [23,24,25].

Microwave heating provides a more rapid and uniform heating than conventional heating, allowing shorter treatment times and more accurate control of the process. Because dielectric heating results from both dipole orientation and ionic conductivity, the addition of salts can increase the heating rate [26]. The rate and yield of carbohydrates and protein solubilization is highly dependent on the conditions, and the presence of salts could also influence the protein extraction [27].

#### 2.2.1. Extraction Yield and Characteristics of the Liquid Fraction

**Figure 2:**
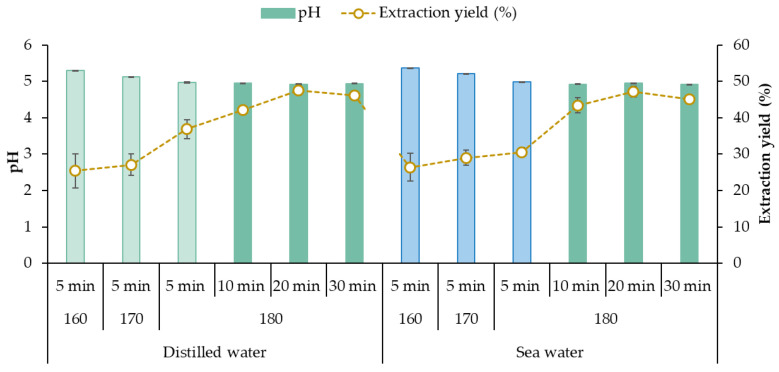
Effect of the processing conditions on the extraction yield and pH of the liquid fraction obtained after microwave-assisted hydrothermal treatment.

The influence of the operation temperature on the extraction yield and composition of the liquid fraction and on the performance of the hydrothermal process was assessed. The solubilization yield increased with temperature at 180 °C (severity 3.4), more markedly when distilled water was used as a solvent (Figure 2). Note here that the severities increased from 2.5 (160 °C) to 3.8 (180 °C). In a recent study using a microwave pretreatment, Fernández-Medina et al. (2022) found maximum solubilization of reducing sugars at higher severity, 220 °C at 20 min, but under those conditions, the monomeric sugars were degraded [17]. In our work, the aim was to explore the possibility of obtaining oligosaccharides, and such elevated temperatures were not considered based on preliminary experiments and previous behavior of other brown seaweeds where hydrothermal treatments at comparable severities were used (2.2 at 160 °C–4.0 at 220 °C) [28]. However, the influence of time was analyzed for the highest tested temperature (180 °C), achieving the highest performance at 20 min without significant differences after 30 min independently of the water used as the solvent agent.

**Figure 3:**
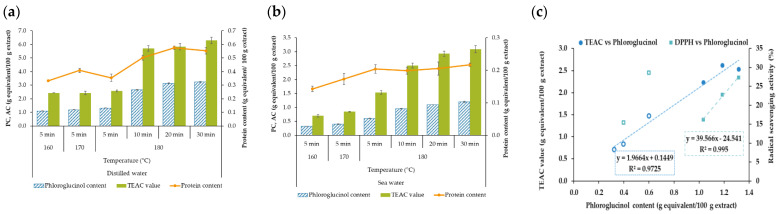
Effect of the processing conditions on the phloroglucinol content (PC), antioxidant activity (AC, such as TEAC and DPPH values), and protein content of the liquid fraction obtained after hydrothermal treatment of *R. okamurae* using different solvents: (**a**) distilled water and (**b**) sea water. (**c**) Correlation between the PC and the AC of the soluble extracts obtained with distilled (filled symbols) and with sea (open symbols) water. Data represent mean ± standard deviation (*n* ≥ 3).

As expected, the total phenolic content of the extracts increased with increasing temperature regardless of the solvent used (Figure 3a,b). Additionally, Fernández-Medina et al. (2022) found this trend and obtained a higher phenolic concentration after microwave-assisted treatment at 200 °C for 20 min [17]. The phenolic extraction yields are equivalent to those reported by those authors. The values attained were lower in the presence of salt water. Other authors found that an increase in salt content involved a negative impact on the recovery of the phenolic compound for different pulses, such as chickpea and beans using microwave-assisted extraction in a saline medium [29]. The loss of total phenolic content from beans during soaking and cooking was also found [30], suggesting that the presence of salts led to the alkylation of the phenolic compounds to other hydroxy propyl by-products. Studies on the stability of phenolic compounds of different families during microwave-assisted extraction indicated an acceleration of the degradation of these fractions during processing [31,32]. Later authors found that those phenolics that have a larger number of hydroxyl-type substituents are more easily degraded under microwave treatment. The influence of the inorganic salts on the hydrolysis of other biopolymers as chitosan in a microwave field implied a promotion of their degradation [33].

The protein content remained under 0.4 g/100 g extract in distilled water and under 0.2 g/100 g extract obtained in sea water, with a slight trend to increase with medium, high temperatures, and time studied. The selected treatment time is low to solubilize the proteins. Note here that waters used as solvent agents presented the following composition: DW (dry content: 47 mg/L; CaCO_3_: n.d.; HCO_3_: <10 mg/L; fluorides: <1 ppm; chlorides: 1 ppm; nitrates: <1 ppm; nitrites: <0.1 ppm; phosphates: <1 ppm; sulfates: <1 ppm) and SW (dry content: 39 g/L; CaCO_3_: n.d.; HCO_3_: <150 mg/L; fluorides: <1 ppm; chlorides: 20,794 ppm; nitrates: <1 ppm; nitrites: <0.1 ppm; phosphates: <1 ppm; sulfates: <2812 ppm). The phenolic compounds seem to be responsible for the antiradical properties (Figure 3c). 

**Figure 4:**
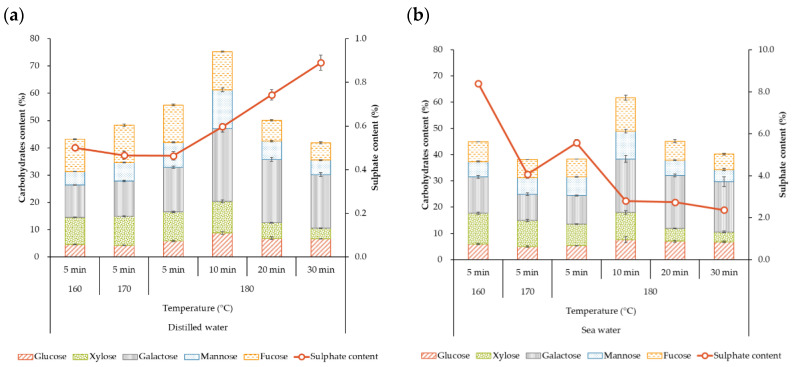
Effect of temperatures and solvents ((**a**) distilled water and (**b**) sea water) during microwave-assisted hydrothermal extraction on the oligosaccharide composition of the liquid fractions obtained from *R. okamurae* and further dialyzed in 0.5 kDa membranes.

The liquid extracts separated after hydrothermal treatment showed no monomeric units. The oligosaccharide content increased with temperature in the range studied for hydrothermal systems treated for 5 min: from 48% to 60% when distilled water was used as solvent and from 38% to 45% when sea water was used (Figure 4). 

The highest oligosaccharide values were identified for liquid extracts treated at 180 °C for 10 min using DW (75%) and SW (62%). The soluble sulfate content was notably lower in distilled water. The low glucose content in the extract could suggest that the cellulose remains in the solid phase. Galactose and fucose were the major monosaccharides. The crude extracts with the highest performance exhibited a Gal:Fuc:Man:Xyl:Glu ratio varying between (1.0:0.5:0.5:0.4:0.3) and (1.0:0.6:0.5:0.5:0.4) for the systems extracted using DW and SW. This behavior is consistent with that reported for crude fucoidans (1.0:0.8:0.1:0.1:0.1) of other Fucales recovered after a pre-treatment with organic solvents followed by a hot water extraction stage [34].

**Figure 5:**
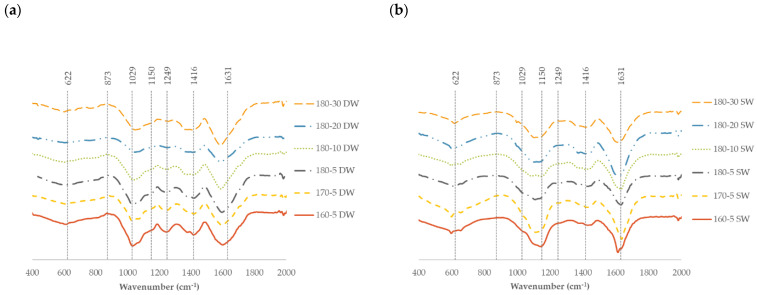
Impact of the MAE extraction treatment in *R. okamurae* brown seaweed using two solvents, (**a**) distilled water (DW) and (**b**) sea water (SW), for the FTIR-ATR spectra of the extracts processed at different temperatures and times.

Data in Figure 5 confirm the presence of sulfate ester bonds at 1249 and 873 cm^−1^ for samples extracted in the presence of distillate water independently of the microwave processing temperature, describing an asymmetrical S=O stretching vibration [35]. All profiles exhibited a major band around 1600 cm^−1^ typically present in the alginate fractions related to the uronic acids. Those recovered using distillate water also presented a band at 1029 cm^−1^ related to the C-O and C-C stretching vibrations of pyranoses, whereas this band was shifted to 1150 cm^−1^ for crude extracts separated using sea water as an extractive agent. The molecular weight of the solubilized polysaccharides is higher than 300 kDa, with the signal from the peaks corresponding to treatments with salt water being more intense with higher molecular weights than those from distilled water treatments (Figure S1).

#### 2.2.2. Cytotoxicity

The extracts obtained with distilled water at 170 °C and at 180 °C showed 49 ± 2% and 43 ± 2% growth inhibition of cell line HeLa 229 at 0.1 mg/mL. The IC_50_ could only be calculated for samples obtained with sea water or with distilled water at 160 °C, which showed a maximum activity with EC_50_= 0.048 mg/mL (Table 2). Note here that extracts obtained after longer hydrothermal treatments (10–30 min) exhibited growth inhibition of cell line HeLa 229 at 0.1 mg/mL below 41% (10 min), 45% (20 min), and 35% (30 min). A comparable activity has been reported for *S. muticum* fucoidans, showing 0.074 mg/mL [36], and activity was lower for those from *U. pinnatifida*, with an IC_50_ of 0.76 mg/mL [37]. These values are in the range of those found for other natural polysaccharides. Additionally, phenolic compounds might contribute to the cytotoxic activity against this cell line [38], but the lyophilized methanolic crude extracts from *Dilophus okamurae* showed weak selective cytotoxic activity to murine L1210 cells and to human leukemic cells, HL60 and MOLT-4 [13].

#### 2.2.3. Alginate Recovery and Characterization

Alginate yield of *Rugulopteryx okamurae* brown alga obtained from the liquid phases after microwave treatment varied between 3.2% and 2.3% for those using distilled and sea water as an extractive agent for 5 min, respectively. The values attained with distilled water were 3.0 ± 0.1%, 3.2 ± 0.1%, and 2.9 ± 0.2% at 160 °C, 170 °C, and 180 °C, respectively. When salt water was used as the solvent, the alginate recovery yields were 2.3 ± 0.1%, 2.5 ± 0.1%, and 2.4 ± 0.1% at 160 °C, 170 °C, and 180 °C, respectively. Therefore, no notable microwave temperature influence in the alginate yield was observed. Note here that there were also no notable differences with increasing extraction time. These low values of calcium alginate are consistent with those previously reported for other invasive brown algae such as *Sargasum muticum* under conventional hydrothermal processing [39].

**Figure 6:**
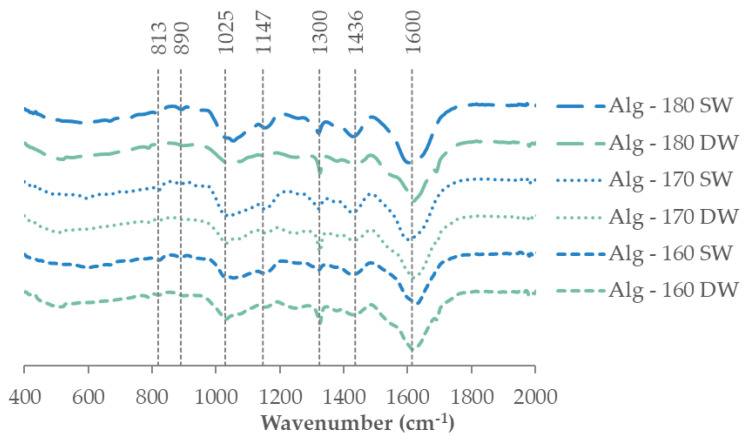
FTIR-ATR spectra for the alginates recovered by calcium chloride precipitation.

FTIR-ATR spectra of the corresponding extracted alginates as representative of recovered biopolymers are presented in Figure 6, exhibiting similar structural features in all cases. Typical signals of the biopolymer are found at 1600 cm^−1^ attributed to the C=O and carboxylate O–C–O asymmetric stretching vibration of uronic acids [40,41]. The absorption bands at 1436 cm^−1^ (C–OH deformation vibration), at 1300 cm^−1^ (C–C–H and O–C–H deformation), at 1147 cm^−1^ (C-O stretching vibrations), and at 1033 cm^−1^ (C-O and C-C stretching vibrations of pyranoses) were also identified. The characteristic weak signals at 890 cm^−1^ (α-L-guluronic asymmetric ring vibration) and at 813 cm^−1^ (β-mannuronic acid) were also observed [42].

Data from ^1^H NMR spectra confirmed the characteristic signals of the extracted alginates allowing the determination of the biopolymer block structure (Table 3) in terms of the individual mannuronic (FM) and guluronic (FG) acids, the M/G ratio, and the four diad frequencies (FMM, FGG, FMG, FGM) [43]. 

The magnitude of FM and M/G rose significantly with rising hydrothermal processing temperature, exhibiting lower values for alginates where sea water was used as the extractive agent. However, M/G ratios varying between 0.47 and 0.82 are consistent with those found in the literature for other brown algae from 0.2 to 1.6 [43,44,45]. The magnitude of this parameter is relevant to have an idea of the rheological features of the extracted biopolymers because those alginates with higher M values tend to present lower viscosities and provide more flexible matrices, whereas those with higher G values involve higher viscosities and more resistant gels [40]. The low values of alternating blocks together with intermediate FGG and low FMM homopolymeric fractions were reported to promote the development of gelled matrices [46].

**Figure 7:**
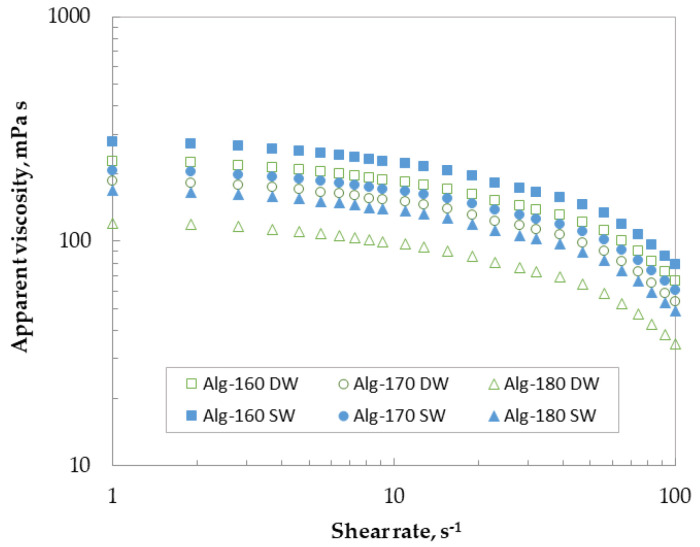
Flow curves for extracted alginates after hydrothermal treatment.

Figure 7 shows the corresponding viscous profiles at 25 °C of the above alginates, extracted after 5 min of hydrothermal treatment, with a commonly used biopolymer content (2%), confirming the hypothesis made with the structural properties. It should be noteworthy that no differences were found with those extracted at higher hydrothermal extraction times. At a fixed shear rate, the apparent viscosity was higher for alginates extracted using sea water than for those processed at the same temperature with distilled water. The highest viscosity values were identified for Alg-160 SW followed by Alg-170 SW (Alg-160 DW) > Alg-180 SW (Alg-170 DW) > Alg-180 DW. At low shear rates, alginate recovered after hydrothermal treatment with both extractive agents showed a Newtonian plateau. In all cases, a shear-thinning behavior was observed above 5 s^−1^. It should be noted that no hysteresis loops were identified in tested alginates. The measured viscosity values were almost half those reported for commercial alginate solutions at similar biopolymer content, but in the range of those found for other crude alginates [39,47].

### 2.3. Characteristics of the Solid Fraction

**Figure 8:**
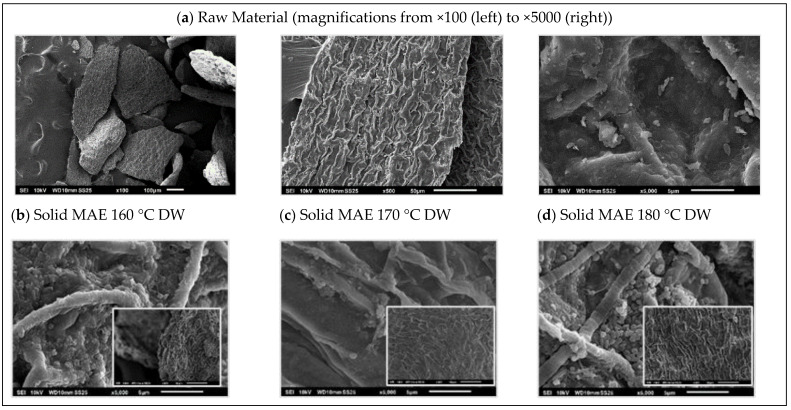

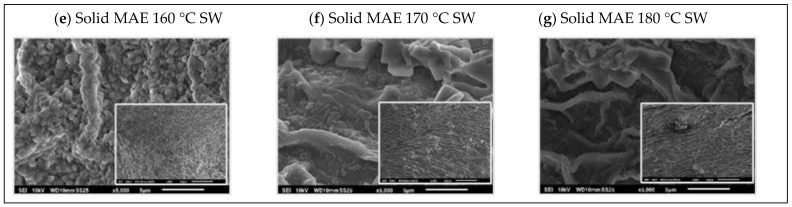
SEM images of *R. okamurae* raw material with different magnifications (**a**) and the corresponding residual solids after microwave-assisted hydrothermal treatment at different processing conditions with distilled water (**b**–**d**) and with salt water (**e**–**g**).

Figure 8 shows representative images of the surface morphology of *R. okamurae* brown alga used as a raw material as well as the corresponding residual solids after hydrothermal treatments at different temperatures in the presence of both extractive agents. The untreated alga presents a regular mosaic-like morphology (Figure 8a–c), which was notably modified during thermal processing. More irregular patterns with larger roughness were identified for the residual solid phases. The presence of small crystalline precipitates from the extraction treatment in those processed in the presence of sea water was also observed. The creation of surface micro-pores and small pieces of debris on the surface suggesting the structural damage has also been reported for milder temperatures (120 °C), enough to enhance the methane production [7].

Nitrogen and carbon contents were higher for solids remaining after extraction with sea water (Table 4). A slight increase was observed with increasing temperature for both extracting solvents. The solid residue was proposed for energetic valorization and for the formulation of bioplastics. Data of the estimated HHV (higher heating value) were slightly higher than for the solid residues remaining after this treatment on a red agarophyte varying between 14,615 and 15,343 kJ/kg [48].

**Figure 9:**
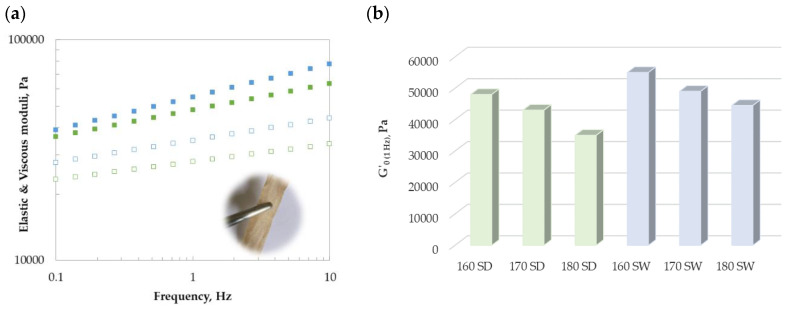
Representative (**a**) viscoelastic profiles and (**b**) variation of G′_0_ (1 Hz) of solid phases of *R. okamurae* blended with glycerol (60/40) after different hydrothermal processing conditions. Symbols: closed (elastic modulus), open (viscous modulus), blue (distilled water), green (sea water).

The potential of solid residue for the development of biomaterials was also studied based on the results previously found for wild *R. okamurae* alga blended with glycerol [18]. Figure 9a shows the viscoelastic profiles obtained for the blends before printing treatment. In all cases, the elastic behavior prevailed over the viscous one, confirming the marked elastic character. A slight frequency dependence was observed in both moduli with a slope around 0.15 ± 0.03 for G′ and about 0.10 ± 0.02 for G″. These dependences are like those previously reported for the blends with the wild seaweed, with an elastic modulus frequency slope of 0.25 before and 0.16 after the heat treatment [18]. Although, the magnitude of both moduli was lower for the blends with the solid residue (about two decades) when compared with the data previously reported for later authors. At a fixed frequency, systems made with solid residues from sea water treatment exhibited higher values than those recovered after distilled water processing (Figure 9b). The strongest viscoelastic features were observed for those prepared with 160 SW followed by 170 SW (160 DW) > 180 SW (170 DW) > 180 DW. These preliminary results evidence the suitability of proposed processing methods for these blends. Further studies are necessary to optimize the mechanical properties of the developed bio-based materials from *R. okamurae* depending on their final application from control–release to food packaging.

**Figure 10:**
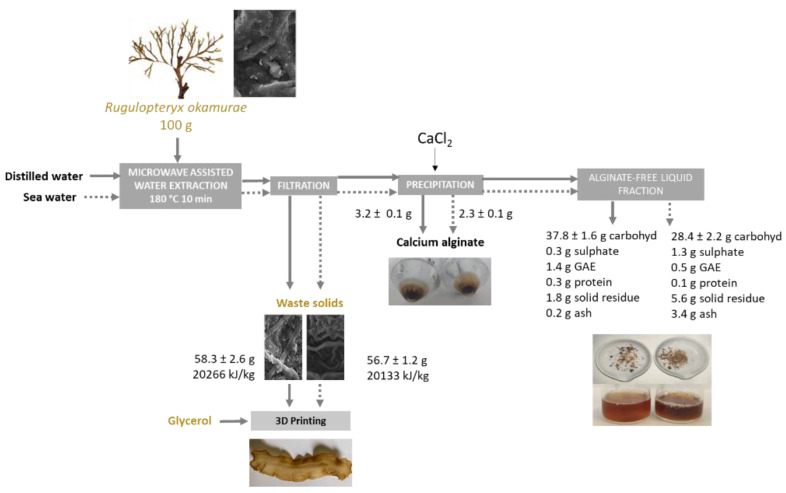
Flow diagram of the proposed process aimed at separating alginate and other bioactive compounds from *R. okamurae* and to formulate novel biopolymers with the residual solids blended with glycerol (60/40) after hydrothermal processing under selected conditions.

Operating under optimal microwave-assisted hydrothermal conditions (180 °C, 10 min), the major seaweed fraction remains insoluble, up to 3 g alginate/100 g seaweed could be obtained by CaCl_2_ precipitation, and a product with up to 75 and 62 g galactofucans/100 g extract could be obtained when distilled and sea water were used, respectively (Figure 10). 

## 3. Materials and Methods

### 3.1. Raw Material

*Rugulopteryx okamurae* was manually collected in July 2021 (Valdevaqueros and Bolonia Beaches, Tarifa, Cádiz, Spain). Algae were separated from extraneous material, washed with tap water, oven dried, ground (≤100 µm), and stored in plastic bags in the darkness at room temperature until use. The sea water was collected at the La Caleta Beach (Cádiz, Spain).

### 3.2. Microwave Assisted Extraction

The extraction was performed using a microwave reactor (Anton Paar Monowave 450, Austria). The ground seaweed was introduced with distilled or sea water in the vials using a solid/liquid ratio of 1:30 (*w*/*w*) following a previous work [37]. The samples were heated until the selected temperature, which was kept for 5 min at 800 rpm and 850 W prior to cooling down to 50 °C. The extraction temperatures were 160 °C, 170 °C, and 180 °C. Longer extraction times (up to 30 min) were assessed at the temperature conditions that led to the best extraction results. Liquid and solid phases were split by vacuum filtration. The solid phases or solid residue were dried and stored at room temperature. 

Severity factor, R_0_, was estimated according to the following equation,

(1) $${\log R}_{0}=log\left\{\left[\int_{0}^{t_{max}}exp\left(\frac{T\left(t\right)-T_{ref}}{\omega }\right)dt\right]+\left[\int_{t_{max}}^{t_{final}}exp\left(\frac{T’\left(t\right)-T_{ref}}{\omega }\right)dt\right]\right\}$$

where *t_max_* (min) is the required time to achieve the target temperature, *t_final_* (min) is the total time for the hydrothermal processing, *T*(*t*) and *T*′(*t*) are the thermal profiles in the heating and cooling steps, respectively, *T_ref_* (100 °C), and *ω* (14.75 °C).

### 3.3. Alginate Precipitation

The alginate fraction was precipitated from the liquid fractions obtained after extraction by MAE using CaCl_2_ (Sigma-Aldrich, St. Louis, MO, USA) at 1% (*w*/*w*) overnight, after the fraction was recovered by centrifugation at 4500 rpm for 40 min (Rotixa 50RS, Hettich Zentrifugen, Tuttlingen, Germany) and dried at 70 °C in the laboratory oven for 48 h. 

### 3.4. Analytical Methods for Raw Material and Solid Residue

Moisture and ash content were gravimetrically determined. In the case of moisture, the raw material was introduced in a laboratory oven for 48 h at 105 °C, and ash content was calculated after calcination of the samples at 575 °C for 6 h in a muffle.

Total nitrogen was analyzed using a Flash EA 1112 Elemental Analyser (Thermo Electron, absorbed for Thermo Fisher Scientific, Waltham, MA, USA) equipped with a multiple analysis column (6 × 5 mm, 2.0 m; Cromlab, Barcelona, Spain). The result was converted to protein using the factor 5.38, specific for brown seaweeds [49].

The content of carbon and hydrogen was determined using an elemental analyzer (Thermo Flash EA 1112, Thermo Fisher Scientific, Waltham, Massachusetts, USA). The operation conditions used for determination were 130 mL/min for helium gas, 100 mL/min for reference, and 250 mL/min for oxygen. The oxidation and reduction temperatures were 900 and 680 °C using a laboratory oven. The analyses were conducted using a column (6 × 5 mm, 2.0 mm) from Cromlab (Spain). The temperature selected was 50 °C and 420 s was selected as the chromatogram time, using aspartic acid as a pattern. Nitrogen (N), hydrogen (H), and carbon (C) content were acquired from the solid phases to determine the higher heating values (HHV) following the equation described elsewhere [48].

The total lipids content was gravimetrically determined after extraction with chloroform:methanol following the Folch method [50]. Briefly, samples were contacted with a chloroform:methanol solution (2:1) in a solid–liquid ratio of 1:20. The mixture was centrifuged for 10 min at 300 rpm and 15 °C. After filtering, 5 mL of distilled water was added, forming a biphasic system that was separated by centrifugation using the same conditions as in the previous stage. The lipid content was calculated after the chloroform (lower phase) was evaporated by rotary evaporation (320 mbar at 40 °C) and dried in an oven (80 °C) for 1 h.

Fatty acid methyl esters (FAMEs) were analyzed by means of transmethylation of lipid samples (10 mg) using a solution (1 mL) of sodium hydroxide (1%) in methanol with heating (55 °C, 15 min). Then, a solution (2 mL) of hydrogen chloride (5%) in methanol (2 mL) (55 °C, 15 min) and water (1 mL) as previously detailed (Carreau and Dubacq, 1978) were added. FAMEs were extracted using hexane prior to evaporation of the organic phase under reduced pressure and measured using a GC-MS Trace GC Ultra (Thermo Fisher, Waltham, MA, USA) with a ZB-WAX column (60 m × 0.25 mm internal diameter × 0.25 µm, Zebron by Phenomenex). 

The minerals of the raw material were analyzed by different methodologies. At first, acidic digestion was performed: 0.3 g ash mixed with 10 mL HNO_3_ and 1 mL H_2_O_2_ were introduced in a Marsxpress (CEM), and the conditions of the protocol used were 1600 W for 15 min, keeping at 200 °C for 10 min. In the case of Ca, Fe, Cu, and Mg, the atomic absorption spectroscopy technique was used, and Na and K were analyzed by atomic emission spectroscopy (spectrometer SpectrAA-220 Fast Sequential from Varian, Palo Alto, CA, USA). Inductively coupled plasma mass spectrometry (ICP-MS, X Series, Thermo Scientific, Waltham, Massachusetts, USA) was used to determine Cd content. 

Extractives were determined using conventional Soxhlet extraction. One part of the ground seaweed was extracted in Soxhlet using organic solvents, ethanol (96%), hexane, and MeOH + Acetone + H_2_O (3:1:1) (Sigma-Aldrich, USA) until the solvent turned colorless.

The oligosaccharide composition of the raw material was performed by HPLC in a 1260 series Hewlett Packard chromatograph (with an IR detector); the column used was an Aminex HPX-87H column (300 × 7.8 mm, BioRad, Hercules, CA, USA) with a pre-guard, operating at 60 °C with 0.003 M H_2_SO_4_ at 0.6 mL/min. The content of oligosaccharides in the raw material was determined after quantitative hydrolysis (72% sulfuric acid at 30 °C, 1 h), after the solution was diluted until a concentration of 2% and introduced in an autoclave at 121 °C for 1 h. Samples were filtered, the liquid phase was analyzed in the HPLC to quantify the oligosaccharide content, the solid phase was introduced in a laboratory oven at 105 °C for 24–48 h, and the residue was gravimetrically quantified as acid-insoluble residue (AIR). 

### 3.5. Analytical Methods for Liquid Samples

The pH of the samples was determined in triplicate with a GLP 21 pH meter (Crison instruments, Barcelona, Spain). 

### 3.6. Phloroglucinol Content

This analysis was performed by spectrophotometry following the protocol described previously [51]. In brief, the liquid sample (1 mL) was introduced in a test tube, and 1 mL of Folin Ciocalteu reagent and 2 mL of Na_2_CO_3_ at 20% were added. The test tube with all the reagents was stirred in a vortex and incubated in darkness at room temperature for 45 min. The standard used was phloroglucinol (Sigma-Aldrich, St. Louis, MO, Spain), performing a standard curve to determine the phloroglucinol content in the samples. The absorbance was measured at 730 nm (Evolution 201 UV–vis, Thermo Scientific, Waltham, MA, USA).

### 3.7. Sulfate Content

The protocol known as gelatin-barium chloride method [52] was performed to spectrophotometrically determine the content of sulfate. Briefly, gelatin-BaCl_2_ reagent was made in two steps: (1) 0.5 g gelatin powder (Scharlau, Barcelona, Spain) was dissolved in 100 mL of distilled water (previously heated until 70 °C), and this solution was kept at 4 °C for at least 6 h (or overnight). After this time, 0.5 g BaCl_2_ (Sigma-Aldrich, Spain) was added, obtaining a cloudy solution, and after 2–3 h, the solution was ready to use. A mixture of 0.2 mL of liquid samples or distilled water (blank), 3.8 mL of trichloroacetic acid solution at 10%, and 1 mL of gelatine-BaCl_2_ reagent was prepared in a test tube and incubated at room temperature for 15 min. The standard curve was performed with potassium sulfate and the absorbance was measured at 500 nm (Evolution 201 UV–vis, Thermo Scientific, USA).

### 3.8. Antioxidant Activity

The trolox equivalent antioxidant capacity (TEAC) value was determined following the protocol described previously to measure the ABTS radical scavenging capacity [53]. At first, the TEAC reagent was prepared: 34.8 mg of ABTS and 6.62 mg of potassium persulfate were dissolved in 10 mL of PBS. The solution was stirred in darkness for 16 h (or overnight). This reagent was diluted with PBS (also used as blank) until the value of 0.7 of absorbance (at 734 nm). The samples or blank (20 μL) and diluted TEAC reagent (2 mL) were mixed in a test tube and incubated at 30 °C for 6 min. The standard curve was performed using trolox (6-hydroxy-2,5,7,8-tetramethylchroman-2-carboxylic acid) as standard. The absorbance was read at 734 nm (Evolution 201 UV–vis, Thermo Scientific, USA). The inhibition percentage was studied following the protocol developed by von Gadow, Joubert, and Hansmann (1997) to scavenge the DPPH radical (α, α-Diphenyl-picrylhydrazyl) [54]. In brief, in a test tube, 2 mL methanolic solution of DPPH at 6 × 10^−5^ M was added above a 50 μL sample solution. The absorbance was read at 10 and 16 min, at 515 nm.

### 3.9. Soluble Protein

The content of soluble protein was analyzed following the protocol known as the Bradford method. The standard curve was performed using Bovine Serum Albumin (BSA, Sigma Aldrich, USA) as standard. Bradford Reagent was used according to the protocol supplied by Sigma-Aldrich. The test tubes were incubated at room temperature for 35 min. The absorbance was read at 595 nm (Evolution 201 UV–vis, Thermo Scientific, USA).

### 3.10. Oligosaccharide Determination

The oligosaccharide content in the hydrolysate samples (previously diafiltered (Spectra/Por Float-A-Lyzer Dialysis Device MWCO: 100–500 Da, SpectrumLabs)) was determined by performing a posthydrolysis with sulfuric acid at 4% at 121 °C in an autoclave (P-Selecta, Spain) for 20 min. The liquid samples were filtrated (0.45 μm, Sartorius, Madrid, Spain) and the determination was performed by High-Pressure Liquid Chromatography (HPLC) using a 1100 series Agilent chromatograph (St. Clara, CA, USA) described above. In this case, the column used was an Aminex HPX87H column (300 × 7.8 mm, BioRad, USA), operating at 60 °C, the mobile phase was 0.003 M H_2_SO_4_ (Sigma-Aldrich, USA), and the flow rate was 0.6 mL/min.

### 3.11. Molar Mass Distribution

High-Performance Size Exclusion Chromatography (HPSEC) was performed to analyze the molar mass distribution of the liquid samples. The column used was a SuperMultipore PW-H column (6 mm × 15 cm) with a guard column SuperMP (PW)-H (4.6 mm × 3.5 cm) from TSKgel by Tosoh Corporation (Japan) operating at 40 °C, fitted in the HPLC described above. The mobile phase was Milli-Q water at 0.4 mL/min. Polyethylene oxide was used as standard, from 23.6 to 786 kDa (Tosoh Corporation, Tokio, Japan).

### 3.12. Fourier-Transform Infrared Spectroscopy

The freeze-dried samples and alginate fractions obtained from MAE were studied by FTIR (Nicolet 6700, source: IR, detector: DTGS KBr). The software used was OMNIC. The extracts were blended with potassium bromide. The spectra were obtained from 400 to 2000 nm, with the resolution of 4 cm^−1^ and 32 scans/min.

### 3.13. Proton Nuclear Magnetic Resonance 

^1^H NMR was performed using a spectrometer (ARX400, Bruker BioSpin GmbH, Germany) for the alginate fractions’ recovery, the concentration of the solutions was 10 mg/mL using deuterated water as solvent and 3-(trimethylsilyl)-L-propane sulfonic acid (Sigma-Aldrich, USA), and the assays were conducted at 75 °C, 400 MHz. For the anomeric protons of the mannuronic (M) and guluronic (G) acids, the signals of 4.70 ppm and 5.08 ppm were found, respectively, and the M/G ratio was estimated.

### 3.14. Cell Inhibition Assay

The cell viability was evaluated using the cervix carcinoma (HeLa 229) cell line. These cells were cultured in DMEM (Dulbeco Modified Eagle’s Medium) and supplemented with FBS (10%) and L-Glutamin (2 mM) and incubated at 37 °C (95% air:5% CO_2_ atmosphere).

The assay performed was the MTT (3-[4,5-dimethylthiazol-2-yl]-2,-5 diphenyltetrazolium bromide) test. HeLa 229 cells were seeded in a 96-well plates with 4000 cells/well and incubated (4–6 h). The extracts were dissolved in Milli-Q water and incubated at 37 °C for 48 h (95% air:5% CO_2_ atmosphere).

After incubation, MTT solution (10 μL) prepared at 5 mg/mL in PBS was added to the well plate and the mixture was incubated (4 h). Finally, SDS dissolved in 0.01 M HCl (100 µL; 10%) was added. Then, an incubation of the well plate for 12–14 h was necessary. The absorbance was read at 595 nm (Tecan Infinite M1000 Pro).

### 3.15. Rheology

The solid phase after hydrothermal treatment was proposed for the development of bioplastics adapted from the procedure previously reported for *Rugulopteryx okamurae* brown seaweed [18]. Following their achievements, the solid phases were blended with glycerol (60/40) at 50 rpm for 15 min before printing on a Foodini 3D printer (Natural Machines, Barcelona, Spain) at 90 °C with a syringe extruder (0.8 mm nozzle) using a rectangular model (60 × 20 × 10 mm). 

Rheological measurements in terms of frequency sweeps (from 0.1 to 10 Hz) were conducted on the blends before and after processing using a controlled stress rheometer (MCR302, Anton Paar Physica, Austria). Note here that the apparent viscosity of extracted alginate solutions at a commonly used biopolymer content (1%) was also measured [55]. Monitoring of the viscoelastic profiles was made by means of a sand-blasted parallel-plate geometry (25 mm diameter, 1 mm gap). Both elastic (G′) and viscous (G″) moduli were recorded at 25 °C within the linear viscoelastic region (15 Pa).

### 3.16. Statistical Analysis

Data were studied using one-factor analysis of variance using the PASW Statistics v.22 software (IBM SPSS Statistics, New York, NY, USA). If means showed differences with 95% confidence (*p* < 0.05), a Scheffé post hoc test was performed.

## 4. Conclusions

To conclude, this study confirms the potential of a chemical-free hydrothermal treatment for the initial fractionation of *Rugulopteryx okamurae* biomass, a stage that could be directly applied to the wet material using sea water. Despite the low yields, alginate with interesting rheological properties can be obtained. The possibility of valorizing the phenolic and the sulfated polysaccharides fraction should be studied. Further purification of bioactive compounds in the water-soluble fraction and exploration of potential uses would be of interest to obtain high value-added products. The residual solids accounted for the major fraction after the microwave-assisted hydrothermal treatment, and exploration of the uses of the biomaterials formulated are encouraged.

## Figures and Tables

**Table 1:** Proximal composition of *Rugulopterix okamurae* in dry basis (*w*/*w*), except moisture content.

Fraction	Content	Metal	Content
Moisture (%)	8.06 ± 0.16	Ca (mg/g)	15.90 ± 1.33
Ash (%)	11.56 ± 0.68	Mg (mg/g)	3.20 ± 0.12
Protein (%)	16.43 ± 0.70	Fe (mg/g)	0.55 ± 0.06
Lipid (%)	6.17 ± 0.15	Na (mg/g)	0.53 ± 0.02
Acid insoluble residue, AIR (%)	30.81 ± 1.00	K (mg/g)	0.40 ± 0.02
**Extractives (%)**		Cu (µg/g)	23.98 ± 0.98
Ethanol (96%)	11.24 ± 0.14	As (µg/g)	5.82 ± 0.40
Hexane	1.73 ± 0.66	Pb (µg/g)	0.97 ± 0.02
MeOH + Acetone + H_2_O (3:1:1)	12.73 ± 0.27	Cd (µg/g)	0.15 ± 0.05
**Carbohydrates (%)**		B (mg/g)	0.041 ± 0.001
Glucose	11.69 ± 0.12	Cu (mg/g)	0.034 ± 0.001
Galactose	2.76 ± 0.07	Hg (µg/g)	0.016 ± 0.001
Fucose	6.38 ± 0.02		
Mannose	1.91 ± 0.05		
Xylose	0.68 ± 0.02		
Acetyl groups	2.36 ± 0.01		
Uronic acids (glucuronic acid eq.)	1.65 ± 0.01		
Sulfate (%)	2.79 ± 0.07		

**Table 2:** Inhibitory efficacy of cell growth (E_max_) and IC_50_ for extracts obtained by MAE in the tumoral cell line HeLa 229.

Samples ^1^	E_max_ (% Inhibition)	IC_50_ (mg/mL)
160 DW	65 ± 2	0.048 ± 0.01
160 SW	60 ± 1	0.689 ± 0.13
170 SW	53 ± 1	0.317 ± 0.02
180 SW	60 ± 3	0.715 ± 0.14
Cisplatin	94 ± 1	0.80 ± 0.03 µM

^1^ Extracts recovered after 5 min of hydrothermal treatment.

**Table 3:** Data from ^1^H NMR spectra of the recovered alginates from microwave-assisted hydrothermal treatment of *R. okamurae*.

Alginate	M/G	F_M_	F_G_	F_MM_	F_GG_	F_MG_
Alg-160 DW	0.56 ^c^	0.36 ^c^	0.64 ^b^	0.25 ^c^	0.53 ^b^	0.11 ^a^
Alg-170 DW	0.69 ^b^	0.41 ^b^	0.59 ^c^	0.29 ^b^	0.47 ^c^	0.12 ^a^
Alg-180 DW	0.82 ^a^	0.45 ^a^	0.55 ^d^	0.69 ^a^	0.42 ^d^	0.13 ^a^
Alg-160 SW	0.47 ^d^	0.32 ^d^	0.68 ^a^	0.24 ^c^	0.60 ^a^	0.08 ^b^
Alg-170 SW	0.58 ^c^	0.37 ^c^	0.63 ^b^	0.28 ^b^	0.54 ^b^	0.09 ^b^
Alg-180 SW	0.69 ^b^	0.41 ^b^	0.59 ^c^	0.30 ^b^	0.48 ^c^	0.11 ^a^

In all cases, standard deviations of the estimated data were <0.01. FMG = FGM. Data values in a column with different superscript letters were significantly different at the *p* ≤ 0.05 level.

**Table 4:** Solid residue after microwave-assisted hydrothermal treatment of *Rugulopteryx okamurae*.

	T (°C)	Nitrogen (%, *w*/*w*)	Carbon (%, *w*/*w*)	Hydrogen (%, *w*/*w*)	HHV (kJ/kg)
Distilled water	160 ^1^	3.28 ± 0.16 ^a^	43.16 ± 0.21 ^c^	5.89 ± 0.30 ^a^	17,508 ± 62 ^f^
	170 ^1^	3.31 ± 0.09 ^a^	44.07 ± 0.08 ^b^	6.10 ± 0.18 ^a^	16,102 ± 188 ^h^
	180 ^1^	3.39 ± 0.01 ^a^	45.44 ± 0.46 ^a^	6.03 ± 0.08 ^a^	17,867 ± 48 ^e^
	180 *	4.10 ± 0.13	46.84 ± 0.50	6.32 ± 0.18	19,123 ± 56 ^c^
	180 **	4.10 ± 0.07	49.58 ± 0.14	6.17 ± 0.15	20,266 ± 98 ^a^
	180 ***	4.11 ± 0.01	49.34 ± 0.13	6.11 ± 0.06	20,141 ± 102 ^a^
Sea water	160 ^1^	2.83 ± 0.04 ^c^	38.94 ± 0.59 ^e^	5.16 ± 0.01 ^b^	16,045 ± 150 ^h^
	170 ^1^	2.86 ± 0.05 ^c^	38.82 ± 0.42 ^e^	5.26 ± 0.05 ^b^	18,413 ± 177 ^d^
	180 ^1^	3.13 ± 0.06 ^b^	41.42 ± 0.28 ^d^	5.43 ± 0.03 ^a, b^	16,900 ± 108 ^g^
	180 *	4.36 ± 0.08	46.80 ± 0.50	6.00 ± 0.11	19,085 ± 121 ^c^
	180 **	4.35 ± 0.17	48.57 ± 0.46	6.11 ± 0.03	19,843 ± 103 ^b^
	180 ***	4.20 ± 0.02	49.19 ± 0.23	6.25 ± 0.08	20,133 ± 99 ^a^

Data are provided as mean ± standard deviation. Values in a column with different superscript letters are statically different (*p* ≤ 0.05). ^1^ Extracts recovered after 5 min of hydrothermal treatment. Microwave extraction time: * 10 min, ** 20 min, *** 30 min.

## Data Availability

The data presented in this study are available on request from the corresponding author.

## Supplementary Materials

The following supporting information can be downloaded at https://www.mdpi.com/article/10.3390/md21060319/s1, Figure S1: Effect of the extraction process in R. okamurae brown seaweed by MAE using two solvents, (a) distilled water (DW) and (b) sea water (SW), for the profiles of molecular weight distribution (in Da) of the extracts processed at different temperatures and times.
