# Supplementary material for: Microwave-Assisted Hydrothermal Processing of Rugulopteryx okamurae
Source: Mar Drugs. 2023 May 25;21(6):319. doi: 10.3390/md21060319 (PMC10304475; doi:10.3390/md21060319)
Supplement: Supplementary file 1 [file marinedrugs-21-00319-s001.zip › marinedrugs-2374427-supplementary.pdf]

a) b)

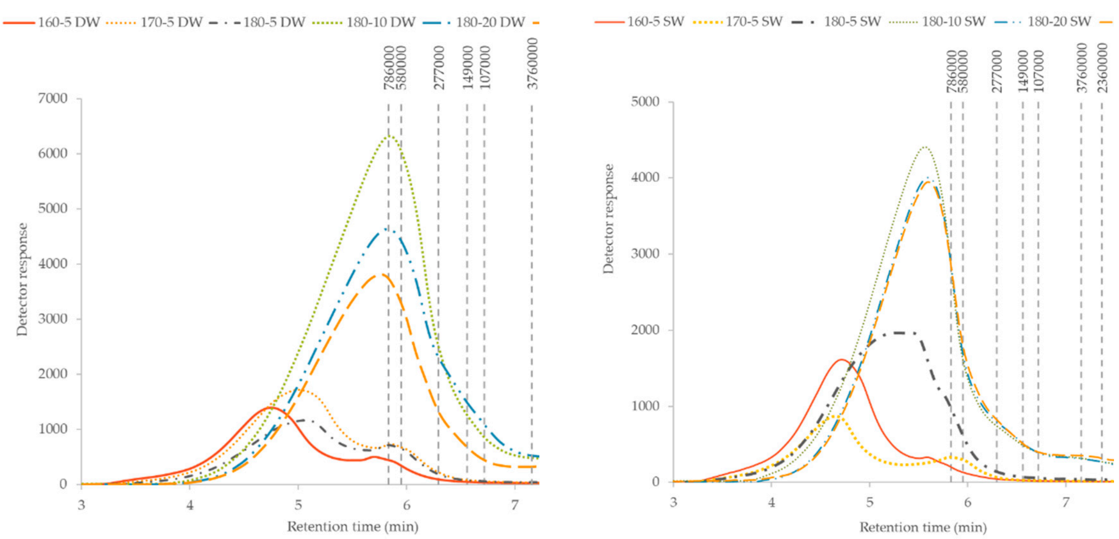


**Figure S1**. Effect of the extraction process in *R. okamurae* brown seaweed by MAE using two solvents (a) distilled water (DW) and (b) sea water (SW) for the profiles of molecular weight distribution (in Da) of the extracts processed at different temperature and time.
